# Porous α-Fe_2_O_3_ Hollow Rods/Reduced Graphene Oxide Composites Templated by MoO_3_ Nanobelts for High-Performance Supercapacitor Applications

**DOI:** 10.3390/molecules29061262

**Published:** 2024-03-12

**Authors:** Gangqiang Zhou, Guo Liang, Wei Xiao, Liangliang Tian, Yanhua Zhang, Rong Hu, Yi Wang

**Affiliations:** 1 School of Materials Science and Engineering, Chongqing University of Arts and Sciences, Chongqing 402160, China; ycfnzgq@163.com (G.Z.); 13512304841@163.com (G.L.); zyhcoco@163.com (Y.Z.); hurong_82@cqwu.edu.cn (R.H.); 2 Institute of Process Engineering, Chinese Academy of Sciences, Beijing 100190, China; wangyi@ipe.ac.cn

**Keywords:** Fe_2_O_3_, reduced graphene oxide, template synthesis, hollow rods, supercapacitor, energy storage

## Abstract

Porous α-Fe_2_O_3_ hollow rods/reduced graphene oxide (α-Fe_2_O_3_ HR/RGO) composites with unique morphological characteristics and a high surface area are prepared through a template strategy, which was systematically studied and found to have outstanding supercapacitive properties. When served as active material in a three-electrode setup, the optimized α-Fe_2_O_3_ HR/RGO-30, comprised 76.5 wt% α-Fe_2_O_3_ and 23.2 wt% RGO, was able to offer the largest specific capacitance of 426.3 F g^−1^, an excellent rate capability as well as satisfactory cycle life with capacitance retention of 87.7% and Coulombic efficiency of 98.9% after continuously charging/discharging at 10 A g^−1^ for beyond 10,000 cycles. Such electrochemical behaviors of the α-Fe_2_O_3_ HR/RGO-30 electrode can rival or even surpass those of many Fe_2_O_3_-based electrodes documented in the previous literature. Later, a symmetric supercapacitor cell of α-Fe_2_O_3_ HR/RGO-30//α-Fe_2_O_3_ HR/RGO-30 was fabricated. The assembled device offers the maximum energy density of 18.7 Wh kg^−1^, and also exhibits commendable rate capability, and features stable cycling durability (with capacitance retention of 83.2% together with a Coulombic efficiency of 99.3% after 10,000-cycle charge/discharge at 5 A g^−1^). These notable electrochemical performances enable the α-Fe_2_O_3_ HR/RGO-30 composite to be a high-potential material for advanced energy storage systems.

## 1. Introduction

Benefiting from conspicuous electrochemical performances such as high-power density, a fast charge/discharge rate, splendid cycling durability, and long lifespan, the supercapacitor has been regarded as one of the most advanced energy storage devices [1,2,3,4,5]. In terms of charge storage mechanisms, supercapacitors are mainly classified into two kinds, which are called electrical double-layer capacitors and pseudo-capacitors [3,4,5,6,7,8,9,10,11]. The former usually depends on carbon-based matters (e.g., porous carbon, graphene, etc.) to accumulate a charge at the electrode/electrolyte interface [3,4,5,6]. The latter stores are charged via reversible Faradaic reactions between electrolyte ions and electroactive materials (e.g., transition metal compounds, conductive polymers, etc.) [9,10,11,12,13,14,15]. Transition metal elements, such as Fe, Ni, Cu, Mo, V, and Mn, are able to provide multiple chemical states for the electrode redox reactions; therefore, their oxides, sulfides, and phosphides are widely explored and studied for supercapacitor applications [10,11,12,13,14,15]. Among them, Fe_2_O_3_ has received considerable attention owing to its high theoretical specific capacitance, suitable potential window, stable properties, natural abundance, and nontoxicity [11,16]. Unfortunately, in bulk form, its low surface area and intrinsic poor conductivity severely limit its electrochemical performance and practical applications. To upgrade the supercapacitive behaviors of Fe_2_O_3_-based materials, two methodologies are usually preferred. One is to construct nanostructures with unique morphologies and abundant porosities. For example, nanosized Fe_2_O_3_ particles [17,18], needles [19], rods [16] and shuttles [20], porous Fe_2_O_3_ nanowires [21] and nanosheets [22], and cube-like and sea urchin-shaped Fe_2_O_3_ superstructures [23,24] were successively developed for supercapacitor electrodes. The other is to couple Fe_2_O_3_ with various conductive species (e.g., graphene [11], carbon nanotube [25], metal nanoparticles [26], MXene [27], etc.) to synthesize the corresponding composites with improved conductivity and enhance the charge storage ability. In spite of much progress, most of the reported Fe_2_O_3_-based electrodes still suffer from the slow transport speeds of electrons and ions during charge/discharge processes, leading to the insufficient utilization of active constituents and more or less bringing about unsatisfactory electrochemical performances.

**Figure 1:**
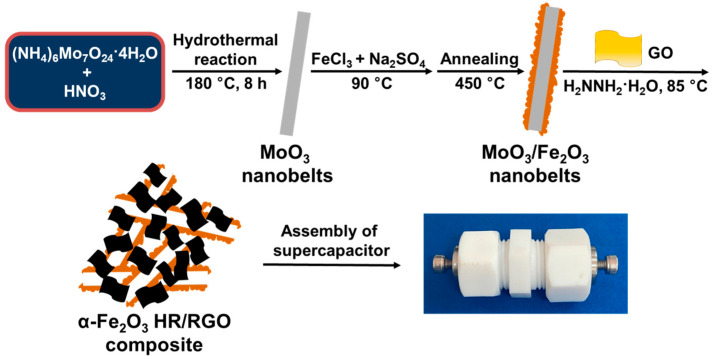
Schematic representation of template-assisted synthesis of α-Fe_2_O_3_ HR/RGO composites for symmetric supercapacitors.

Rational designed hollow structures with mesoporous characteristics may provide sufficient contact between electrode materials and the electrolyte and shorten the ionic diffusion distance. Template synthesis has been proven to be a facile and effective way to fabricate Fe_2_O_3_ hollow materials. For instance, multi-shelled hollow Fe_2_O_3_ microspheres [28], hollow Fe_2_O_3_ microboxes [29], and carbon fabric-supported Fe_2_O_3_ nanotubes [30] have been well developed employing carbonaceous microspheres, Prussian blue microcubes and ZnO nanowires as templates, respectively. On the other hand, to prevent serious aggregation and elevate conductivity, graphene is an ideal candidate for hybridization with metal oxides due to its two-dimensional-layered structure, fantastic conductive properties, and splendid structural stability [11]. The resulting metal oxide/graphene composites often show great improvement in electrochemical properties compared to bare metal oxides. Therefore, based on the above strategies, herein, MoO_3_ nanobelts are hydrothermally synthesized as substrates for the uniform deposition of α-Fe_2_O_3_ nanoparticles through a convenient solution method. Subsequently, the resulting core–shell-structured MoO_3_/α-Fe_2_O_3_ nanobelts undergo treatment with hydrazine monohydrate in the presence of graphene oxide (GO). During this reaction, the total removal of the MoO_3_ template, the conversion of GO to reduced graphene oxide (RGO), and the hybridization of RGO with α-Fe_2_O_3_ simultaneously take place in one pot, yielding porous α-Fe_2_O_3_ hollow rods/RGO (denoted as α-Fe_2_O_3_ HR/RGO) composites (Figure 1). Thanks to the well-developed porosity, well-defined hollow structure, large surface area as well as effective synergy between the two constituents, the currently synthesized α-Fe_2_O_3_ HR/RGO composites exhibit superior supercapacitive properties in both three- and two-electrode systems compared to other reported Fe_2_O_3_-based electrode materials, showing remarkable charge storage advantages.

## 2. Results and Discussion

### 2.1. Materials Characterizations

**Figure 2:**
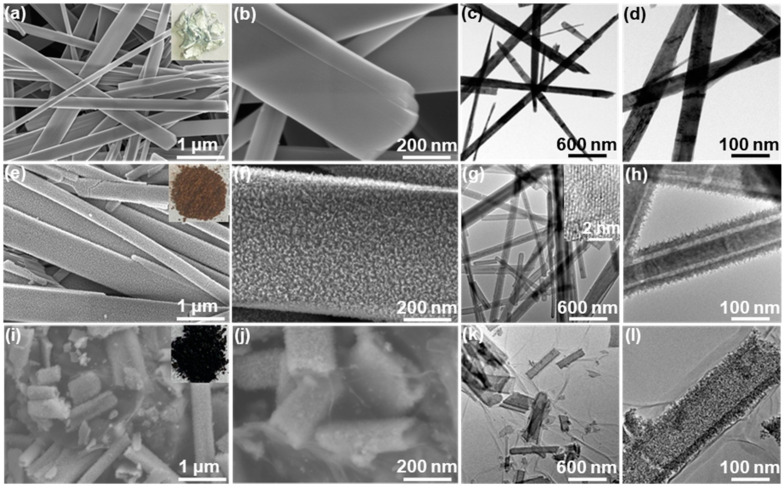
(**a**,**b**) FESEM and (**c**,**d**) TEM images of MoO_3_ nanobelts; the inset in (**a**) is a digital picture of the specimen. (**e**,**f**) FESEM and (**g**,**h**) TEM images of MoO_3_/α-Fe_2_O_3_ composite nanobelts; the insets in (**e**,**h**) are an optical photograph of the sample and HRTEM image of a random α-Fe_2_O_3_ nanoparticle deposited on the external surface, respectively. (**i**,**j**) FESEM and (**k**,**l**) TEM images of the α-Fe_2_O_3_ HR/RGO-30 composite at different magnifications; the inset in (**i**) is its digital picture.

The inset of Figure 2a is a digital picture of hydrothermally synthesized MoO_3_ nanobelts, showing white in color. In terms of the FESEM and TEM observations in Figure 2a–d, MoO_3_ nanobelts have a smooth outer surface with lengths ranging from several to several tens of micrometers and the width between several tens and several hundreds of nanometers. The color of MoO_3_/α-Fe_2_O_3_ composite nanobelts turns dark brown, as presented in the inset of Figure 2e, and their external surface becomes quite rough (Figure 2e,f). Such color transition and surface roughness should be attributable to the uniform deposition of Fe_2_O_3_ on the MoO_3_ substrate. Furthermore, as revealed by the TEM examination in Figure 2g,h, the deposited numerous small α-Fe_2_O_3_ nanoparticle pile adheres together to form a ~30-nm-thick porous shell on each MoO_3_ nanobelt, leading to the unique core–shell structure of the sample. A high-resolution TEM image of an individual α-Fe_2_O_3_ nanoparticle is given in the inset of Figure 2g, where lattice fringes (d-spacing = 0.25 nm) are clearly visible, agreeing well with that of the (110) plane of α-Fe_2_O_3_ [11,19,30]. The inner core of MoO_3_/α-Fe_2_O_3_ nanobelts was selectively dissolved by hydrazine in the presence of GO. During such a reaction, GO was reduced to RGO, which was simultaneously hybridized with the resulting α-Fe_2_O_3_, thereby producing a black α-Fe_2_O_3_ HR/RGO composite (inset of Figure 2i). Such results were identified by the morphological inspections of the α-Fe_2_O_3_ HR/RGO-30 composite (Figure 2i–l). Clearly, the hierarchical porous features and the thickness of the shell are exactly inherited from MoO_3_/α-Fe_2_O_3_ nanobelts. RGO sheets are free from serious aggregation and restacking. In addition, there is a well-defined interior cavity in each α-Fe_2_O_3_ HR, which is also intertwined or wrapped with crumpled RGO sheets, demonstrating the complete removal of the sacrificial template and the sufficient combination of the two components. However, the length of α-Fe_2_O_3_ HR within α-Fe_2_O_3_ HR/RGO-30 composite seems much shorter than that of MoO_3_ nanobelts. It is assumed that such a fact should arise from the fracture of a template during hydrazine-involved processes.

**Figure 3:**
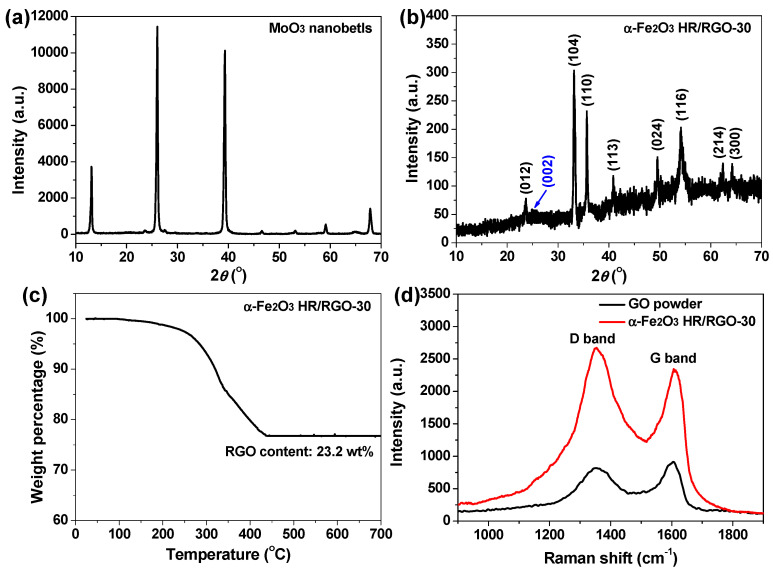
(**a**,**b**) XRD patterns of MoO_3_ nanobelts and α-Fe_2_O_3_ HR/RGO-30 composite, respectively. (**c**) TGA curve of α-Fe_2_O_3_ HR/RGO-30 composite. (**d**) Raman spectra of GO powder and α-Fe_2_O_3_ HR/RGO-30 composite.

The XRD technique was utilized to analyze the crystalline structures of the synthesized materials. The XRD pattern of MoO_3_ nanobelts presents several clear sharp peaks with high diffraction intensity (Figure 3a), matching up with the JCPDS No. #47-1320 for monoclinic MoO_3_ [10,31]. However, these strong peaks cannot be found in the XRD pattern of the α-Fe_2_O_3_ HR/RGO-30 composite (Figure 3b), once again indicating the total depletion of the MoO_3_ template during the synthetic process. Additionally, eight typical peaks located at 2θ = 23.8°, 33.1°, 35.5°, 40.8°, 49.5°, 54°, 62.4° and 64.1° in the XRD pattern of the α-Fe_2_O_3_ HR/RGO-30 composite were well indexed to the (012), (104), (110), (113), (024), (116), (214) and (300) planes of the crystallized hematite structure (JCPDS No. #33-0664), respectively [11,29,30], while another broad weak peak centered around 2θ = 25° (pointed out by the blue arrow) was ascribed to the (002) reflection of RGO [11,30,32], suggesting the effective composition of the two components and no other impurities. The TGA measurement was implemented to ascertain the mass content of RGO within the sample. As depicted in Figure 3c, the TGA curve of the α-Fe_2_O_3_ HR/RGO-30 composite shows a steep decrease from 300 to 450 °C on account of the combustion of RGO in air. As a result, the weight percentages of RGO and Fe_2_O_3_ were readily obtained, which were 23.2 and 76.5 wt%, respectively. The Raman spectra of the GO and α-Fe_2_O_3_ HR/RGO-30 composite are displayed in Figure 3d, where two main bands appear in each curve. The band centered around 1350 cm^−1^ (D band) is associated with the A_1g_ vibration mode of disordered carbon, while the other band centered around 1600 cm^−1^ (G band) corresponds to the E_2g_ vibration mode of an ordered in-plane sp^2^ carbon atom network [11,30,32,33]. As documented in the literature, the intensity ratio of the D to G band (I_D_/I_G_) can be regarded as a measure to determine the structural disorder degree of graphene [11,34,35]. That is, a higher I_D_/I_G_ value means more defects and edges [11,34,35]. The I_D_/I_G_ value for the α-Fe_2_O_3_ HR/RGO-30 composite (1.14) is apparently larger than that for GO powder (0.9), illustrating the conversion of GO to RGO during the hydrazine-involved reductive process, which was spontaneously hybridized in the final product.

**Figure 4:**
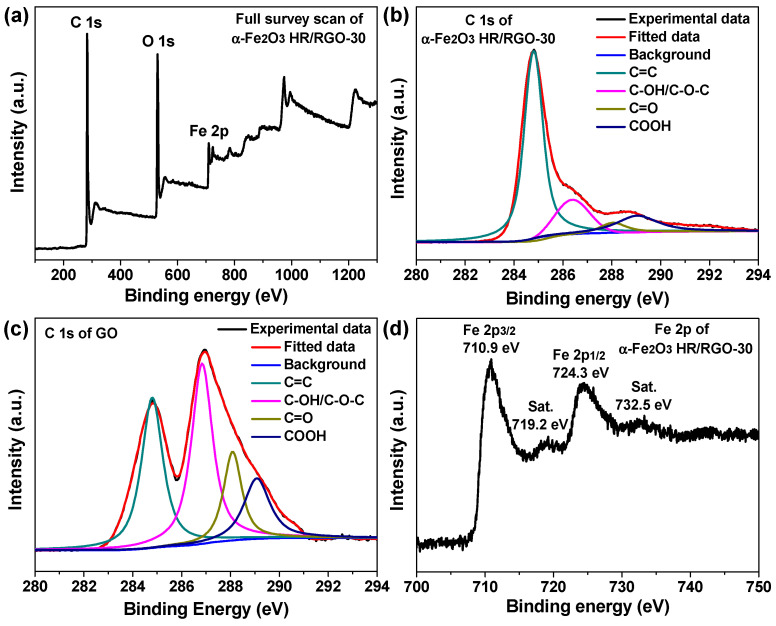
(**a**,**b**) Full-survey-scan and C 1s XPS spectra of α-Fe_2_O_3_ HR/RGO-30 composite. (**c**) C 1s XPS spectrum of GO powder. (**d**) High-resolution XPS spectrum of α-Fe_2_O_3_ HR/RGO-30 composite for the Fe 2p region.

To examine the surface chemical states of the corresponding elements within the α-Fe_2_O_3_ HR/RGO-30 composite, XPS analysis was carried out. Figure 4a reveals its full-survey-scan spectrum, and three kinds of element information for C, O and Fe were detected as envisioned. Figure 4b,c presents the deconvoluted high-resolution C 1s spectra of the α-Fe_2_O_3_ HR/RGO-30 composite and GO powder, both of which can be approximately divided into four peaks. The peak emerging at 284.8 eV can be assigned to the presence of C=C bonding in aromatic rings, whereas the other three peaks centered at 286.4, 288.1, and 289.1 eV, are related to some oxygen-containing groups, such as C–OH and C–O–C, C=O, and COOH [36,37,38]. The relative intensity of oxygen-free bonding to oxygen-containing bonding for the α-Fe_2_O_3_ HR/RGO-30 composite is much smaller than that for GO powder, once again confirming its sufficient chemical reduction to RGO [26,38,39]. Figure 4d is the high-resolution Fe 2p spectrum of the α-Fe_2_O_3_ HR/RGO-30 composite, where a pair of dominant peaks, corresponding to Fe 2p_3/2_ and Fe 2p_1/2_ spin orbitals, are found at 710.9 and 724.3 eV, respectively [19,29,40,41]. In addition, two weak shake-up satellite peaks (marked as Sat.) appear at positions 719.2 and 732.5 eV as well. These binding energy values suggest that the Fe species incorporated in this sample owns the chemical oxidation state of +3 and exists in the form of Fe^3+^ [19,29,40,41]. All the XPS data and analyses also coincide with the above XRD and Raman results.

**Figure 5:**
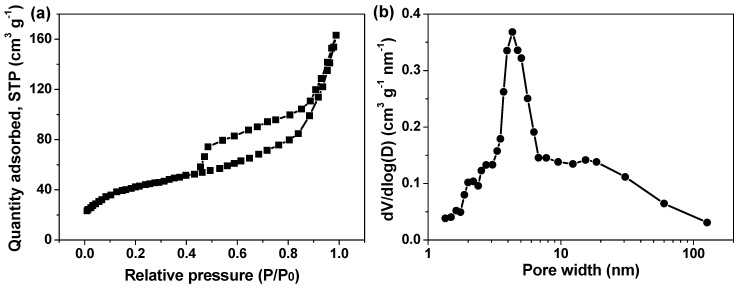
(**a**) Nitrogen adsorption/desorption isotherm curve and (**b**) pore size distribution diagram of α-Fe_2_O_3_ HR/RGO-30 composite.

The porous nature of α-Fe_2_O_3_ HR/RGO-30 was characterized by the Brunauer–Emmertt–Teller (BET) method. A type-IV curve with a clear hysteresis loop at a relative pressure of 0.44–1.0 was obtained from the nitrogen adsorption/desorption isotherm (Figure 5a), implying the presence of a mesoporous structure in the composite [11,29,42]. As further proven in Figure 5b, most of the pores indeed distribute in the mesoporous range (2–50 nm) accompanied by a few micropores (˂2 nm) and macropores (>50 nm). It is believed that the well-developed porosity mainly originates from the growth, accumulation, and agglomeration of numerous Fe_2_O_3_ nanoparticles to form hollow-structured porous Fe_2_O_3_ nanorods. The estimated specific surface area of the α-Fe_2_O_3_ HR/RGO-30 composite is 168.6 m^2^ g^−1^, which remarkably exceeds that of Fe_2_O_3_-based compounds and composites reported previously, including α-Fe_2_O_3_ nanowires (70.6 m^2^ g^−1^) [21], α-Fe_2_O_3_@Ag microboxes (128 m^2^ g^−1^) [29], V_2_O_5_-doped α-Fe_2_O_3_ nanotubes (95.9 m^2^ g^−1^) [43], α-Fe_2_O_3_ nanoplates/the RGO composite (38.04 m^2^ g^−1^) [44] and the Fe_2_O_3_ nanoparticles/MXene composite (14.5 m^2^ g^−1^) [45]. The ample porous architecture, superior surface area, and unique morphology can endow the α-Fe_2_O_3_ HR/RGO-30 composite with abundant active sites for electrochemical redox reactions and, meanwhile, might be in favor of the impregnation and diffusion of electrolyte ions through pore channels and voids [11,21,44]. As a consequence, efficient contact between the electrode and electrolyte, with the easy and fast access of ions, is expected to realize the significant enhancement of the charge storage ability of the α-Fe_2_O_3_ HR/RGO-30 composite.

### 2.2. Electrochemical Evaluation

**Figure 6:**
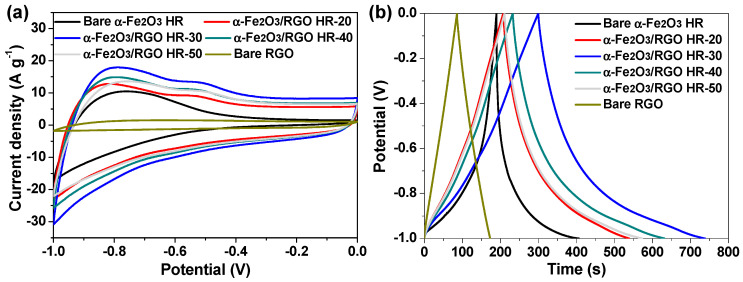
Comparisons of electrochemical behaviors among bare RGO, bare α-Fe_2_O_3_ HR and a series of α-Fe_2_O_3_ HR/RGO composite electrodes. (**a**) CV curves at 50 mV s^−1^. (**b**) GCD curves at 1 A g^−1^.

**Figure 7:**
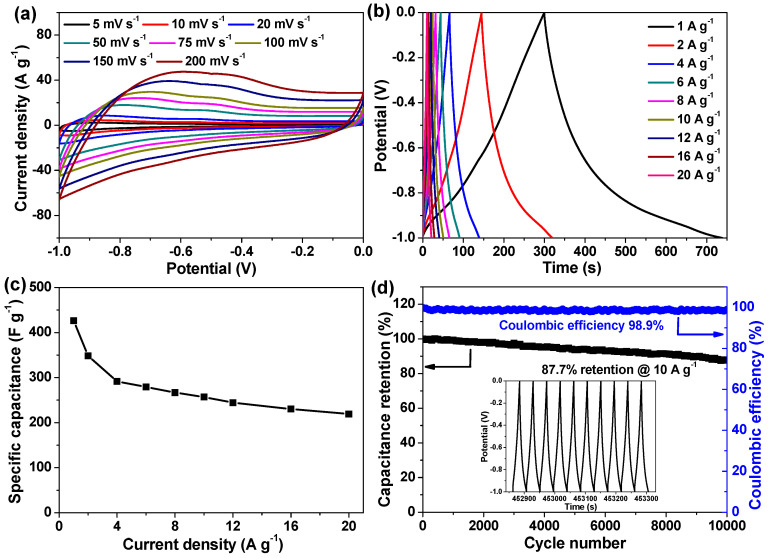
Electrochemical behaviors of α-Fe_2_O_3_ HR/RGO-30 electrode in a three-electrode setup. (**a**) CV curves at various sweep rates. (**b**) GCD curves at different current densities. (**c**) Relationship of specific capacitance with respect to current density. (**d**) Performance of cycling stability experiment; the inset presents the last ten-cycle charge/discharge curves for such tests.

To figure out the advantages of the currently developed materials for supercapacitor applications, the electrochemical properties of bare RGO, bare α-Fe_2_O_3_ HR, and a series of α-Fe_2_O_3_ HR/RGO composite electrodes were studied by employing a standard three-electrode system. Figure 6a compares the corresponding CV curves at the identical sweep rate. The current response and the area enclosed by the CV curve of the α-Fe_2_O_3_ HR/RGO-30 electrode is evidently higher than those of other electrodes, indicating the largest specific capacitance [26,34,42]. Such a result is also testified based on the GCD measurements at a constant current density (Figure 6b) because the discharge duration of the α-Fe_2_O_3_ HR/RGO-30 electrode is the longest among all tested electrodes [26,34,42]. Therefore, the α-Fe_2_O_3_ HR/RGO-30 composite was selected for the following systematic investigation and assembly of the supercapacitor device. Figure 7a illustrates its CV curves at varied scan rates. It can be seen that the CV curves deviate from an ideal rectangular shape to some extent and possesses a wide Faradaic hump, which is indicative of the dominant pseudocapacitive characteristics [29,42]. With a considerable increase in the sweep rate from 5 to 200 mV s^−1^, the CV curves generally preserve a similar shape, suggesting the satisfactory reversible redox process and commendable rate capability [11,29,34]. Figure 7b is a set of GCD curves of the α-Fe_2_O_3_ HR/RGO-30 electrode. The distortion of linearity for the charge and discharge parts demonstrates that pseudo-capacitance makes the main contribution to charge storage. Such a fact is also consistent with the CV results. Moreover, there is only a small potential drop in all GCD curves, reflecting the low internal resistance of this electrode [26,30]. In terms of Equation (1), the specific capacitance was calculated to be 426.3, 348.3, 291.7, 279.2, 266.6, 256.8, 244.4, 230.5 and 219.1 F g^−1^ at 1, 2, 4, 6, 8, 10, 12, 16 and 20 A g^−1^, respectively. These data are depicted in Figure 7c. Impressively, the specific capacitance of the α-Fe_2_O_3_ HR/RGO-30 electrode at 20 A g^−1^ was 51.4%, as much as that at 1 A g^−1^, exhibiting prominent rate performance. Cycle life is known as another crucial parameter for energy storage materials. The α-Fe_2_O_3_ HR/RGO-30 electrode was evaluated using the repetitive GCD test at a large current density of 10 A g^−1^ for over 10,000 cycles. As vividly observed in Figure 7d, the capacitance fading is quite slow, with a final retention of 87.7%, and the Coulombic efficiency of ~100% was also achieved from beginning to end. During the consecutive charge/discharge process, the structural and morphological change, as well as the exfoliation of a small amount of electrode material from the current collector, might be responsible for the gradual capacitance decay [23,43]. It is noteworthy noting that the last 10-cycle charge/discharge curves could maintain a good shape as well (inset of Figure 7d). Moreover, a comprehensive comparison of electrochemical properties between the currently developed α-Fe_2_O_3_ HR/RGO-30 composite and some reported excellent Fe_2_O_3_-based materials was made. Based on the data of maximum specific capacitance, rate capability, and cyclic durability listed in Table 1, the α-Fe_2_O_3_ HR/RGO-30 composite was able to rival or even outperform most of the others, confirming the outstanding charge storage advantages. It is assumed that three main reasons can account for the superior supercapacitive properties. Firstly, the intimate combination of α-Fe_2_O_3_ HR with highly conductive RGO sheets not only prevents them from severe aggregation/restacking but also significantly improves the electrical conductivity of the composite, thus facilitating charge transfer and accelerating the Faradaic redox reaction during the rapid charge/discharge process. Secondly, the hierarchical porous structure with a large surface area can ensure plenty of available active sites for charge storage, thereby making full use of the electrode material. Thirdly, the interior cavities of α-Fe_2_O_3_ HR may serve as electrolyte ion reservoirs, which could shorten their migration distance and favor their diffusion within the electrodes to a large extent.

**Figure 8:**
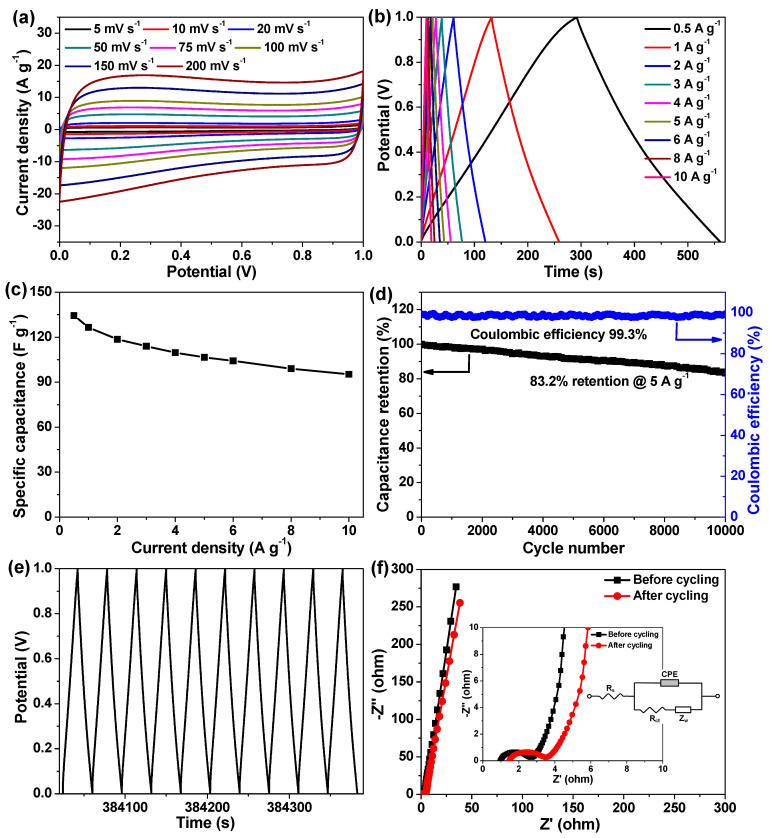
Electrochemical performances of the symmetric α-Fe_2_O_3_ HR/RGO-30//α-Fe_2_O_3_ HR/RGO-30 supercapacitor cell. (**a**) CV curves measured at scan rates of 5–200 mV s^−1^. (**b**) GCD curves tested at current densities of 0.5–10 A g^−1^. (**c**) The relationship of specific capacitance versus current density. (**d**) The results of the cycle durability experiment. (**e**) Charge/discharge curves for the last ten cycles. (**f**) Pre- and post-cycling Nyquist plots; insets are the magnified view for the high-frequency region and the equivalent circuit model.

Apart from the tests in a three-electrode setup, we further constructed a symmetric supercapacitor cell using the α-Fe_2_O_3_ HR/RGO-30 composite as an active electrode material to evaluate the supercapacitive properties at an output potential difference of 0–1 V. As presented in Figure 8a,b, all the CV curves possess an analogous quasi-rectangular shape with acceptable symmetry and all the GCD curves display a nearly isosceles triangular shape, suggesting that such supercapacitor cells feature excellent capacitive behaviors and electrochemical reversibility [24,29,41]. Specific capacitance is deduced from the GCD curves, and the related data are plotted in Figure 8c, with the highest one obtained at 0.5 A g^−1^ with a value of 134.4 F g^−1^. Impressively, the supercapacitor cell exhibits a satisfactory rate performance since the specific capacitance remains as much at 95.2 F g^−1^ by booting the current density even up to 10 A g^−1^. The cycling stability of this device was investigated by repetitive GCD measurements as well. As shown in Figure 8d, after consecutively charging/discharging at 5 A g^−1^ for more than 10,000 cycles, a capacitance decay of only 16.8% and Coulombic efficiency of close to 100% was realized. In addition, the last 10-cycle charge/discharge curves still stayed as well-shaped triangles (Figure 8e), convincingly demonstrating stable electrochemical performances. The EIS spectra of the supercapacitor cell before and after cycling were recorded, and the Nyquist diagrams are depicted in Figure 8f, displaying a similar shape with a small arc in the high-frequency section as well as an abrupt line in the low-frequency section. Both of the two Nyquist plots could be fitted with the same analog circuit, where R_s_, R_ct_, Z_w_, and CPE referred to the overall internal resistance, charge transfer resistance, Warburg impedance, as well as the constant phase element (inset of Figure 8f), respectively [24,29]. Before cycling, the R_s_ and R_ct_ were 0.97 and 1.71 Ω, respectively, and such low values unraveled the rather limited internal resistance, remarkable conductivity, and fast charge transfer rate of this supercapacitor [24,26,34]. After cycling, R_s_ and R_ct_ only exhibited a limited change, with the values only increasing to 1.46 and 2.09 Ω, respectively, once again indicating outstanding long-term cycling durability.

**Figure 9:**
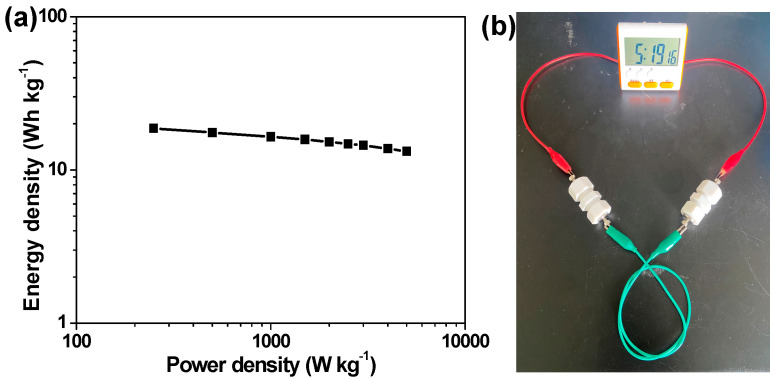
(**a**) Ragone plot of the currently developed α-Fe_2_O_3_ HR/RGO-30//α-Fe_2_O_3_ HR/RGO-30 supercapacitor cell. (**b**) Digital photograph of a portal timer well-powered by two α-Fe_2_O_3_ HR/RGO-30//α-Fe_2_O_3_ HR/RGO-30 supercapacitor cells connected in series.

The energy and power densities of α-Fe_2_O_3_ HR/RGO-30//α-Fe_2_O_3_ HR/RGO-30 supercapacitor devices are calculated according to Equations (3) and (4), and the corresponding data are profiled in the Ragone plot (Figure 9a). Typically, it delivers the maximum energy density of 18.7 Wh kg^−1^ at a power density of 250 W kg^−1^ and retains an energy density of 13.2 Wh kg^−1^ at a large power density of 5000 W kg^−1^. Finally, two α-Fe_2_O_3_ HR/RGO-30//α-Fe_2_O_3_ HR/RGO-30 supercapacitor cells were connected in series and fully charged with the whole voltage of 2 V as a power source to examine practical feasibility and usefulness. As exhibited in Figure 9b, a portable timer was successfully driven by such a tandem device, manifesting the satisfactory charge storage ability and commendable application potential.

## 3. Materials and Methods

### 3.1. Chemicals

FeCl_3_·6H_2_O, (NH_4_)_6_Mo_7_O_24_·4H_2_O, KOH, Na_2_SO_4_, concentrated HNO_3_ (65 wt%), hydrazine monohydrate (80 wt%), absolute ethanol, polyvinylidene fluoride, acetylene black, and *N*-methyl-2-pyrrolidone (NMP) were bought from J&K Co., Ltd. (Shanghai, China). Nickel foam and GO powder were provided by Shenzhen Suiheng Co., Ltd. (Shenzhen, China).

### 3.2. Fabrication of α-Fe_2_O_3_ HR/RGO

In total, 1.2 g of (NH_4_)_6_Mo_7_O_24_·4H_2_O and 40 mL of concentrated HNO_3_ were dissolved in 200 mL of water together. The resulting solution was put into an autoclave with a 0.5 L volume capacity. The reaction proceeded at 180 °C for 8 h in an oven to produce MoO_3_ nanobelts, which were isolated and dried. Next, 60 mg of MoO_3_ nanobelts were dispersed in 60 mL of aqueous solution containing 56 mg of Na_2_SO_4_ and 108 mg of FeCl_3_·6H_2_O. The resulting mixture was continuously stirred and heated to 90 °C, which allowed for a reaction for 2 h. Then, the solid product was harvested, dried, and annealed at 450 °C for 2 h in the air to give MoO_3_/α-Fe_2_O_3_ nanobelts. Afterwards, 100 mL of aqueous suspension containing 160 mg of MoO_3_/α-Fe_2_O_3_ was mixed with x mL (x = 20, 30, 40, and 50) of GO dispersion (2 mg mL^−1^), followed by the introduction of 2 mL of hydrazine monohydrate (80 wt%) under stirring. The reaction was carried out at 85 °C for about 60 min. Due to the presence of hydrazine, the reaction system was alkaline, leading to the etching of the MoO_3_ template. Meanwhile, GO was chemically reduced to RGO by hydrazine during such a process, which was coupled with α-Fe_2_O_3_ to yield the α-Fe_2_O_3_ HR/RGO composite. The final product was collected, washed, and freeze-dried for further use and was labeled as α-Fe_2_O_3_ HR/RGO-x. As a control, bare α-Fe_2_O_3_ hollow rods (labeled as α-Fe_2_O_3_ HR) and RGO were also synthesized in the absence of either GO or MoO_3_/α-Fe_2_O_3_ according to the above procedure.

### 3.3. Characterizations

Field emission scanning electron microscope (FESEM) examinations were performed on Zeiss Gemini SEM 300 (Jena, Germany) with an acceleration voltage of 5 kV, and the specimens were prepared by directly coating the corresponding powder on conductive carbon tape. Transmission electron microscope (TEM) inspections were performed on FEI Tecnai G2 F20 (FEI, Hillsboro, OR, USA) with an acceleration of 200 kV, and the specimens were prepared by dispersing the corresponding powder in absolute ethanol, followed by dropping a small amount of suspension on a carbon-coated copper mesh grid. X-ray diffraction (XRD) patterns were obtained on the Tongda TD-3500 diffractometer (Dandong Tongda Science&Technology Co., Ltd., Dandong, China) employing Cu K_α_ (λ = 0.15405 nm) as radiation sources, and the scan range of 2θ was between 10° and 70° with a step of 0.03°. Raman spectra were collected on Bruker RFS-100 spectrometer (Billerica, MA, USA) in the wavenumber range from 800 to 2000 cm^−1^ with a spectral resolution of 2 cm^−1^. The spot size and wavelength of the exciting laser beam were ~2 μm in diameter and 514 nm, respectively. Thermal gravimetric analysis (TGA) was implemented in the air on TA Instruments SDTQ600 (New Castle, DE, USA) at a heating rate of 10 °C min^−1^ from room temperature to 700 °C. N_2_ adsorption/desorption experiments were conducted at a liquid N_2_ temperature of −196 °C on Micrometrities ASAP 2020 (Norcross, GA, USA) with the weight of the sample more than 200 mg. The specific surface area was estimated based on the Brunauer–Emmett–Teller (BET) model, and the pore size distribution was acquired according to the original density functional theory combined with non-negative regularization and medium smoothing. X-ray photoelectron spectroscopy (XPS) measurements were taken on Perkin Elmer PHI5300 (Waltham, MA, USA), with the Al K_α_ (hυ = 1486.6 eV) radiation source working at a voltage of 12 kV under residual pressure less than 5 × 10^−7^ mbar. The analyzed area of each sample was 400 μm in diameter with the analyzed depth within 10 nm. Energy steps during XPS data collection were 1.0 and 0.05 eV for the full survey scan and high-resolution scan, respectively. The position calibration of XPS peaks was referenced to the binding energy of the C 1s peak at 284.8 eV. Moreover, XPS data fitting was accomplished using software named XPSPeak v4.1 with the adoption of mixed Gaussian–Lorentzian functions and Shirley’s background.

### 3.4. Electrochemical Performance Evaluation

The working electrodes were fabricated as follows. Active materials, acetylene black, and polyvinylidene fluoride were mixed together in accordance with a mass ratio of 70:20:10, and then moderate solvent (NMP) was dropwise-added into the mixture, followed by gentle grinding to form a homogeneous slurry. Nickel foam was cut into rectangular (1 cm × 3 cm in size) and circular (1.1 cm in diameter) shapes. Subsequently, using a painting brush, the appropriate slurry was uniformly loaded on several pieces of rectangular and circular nickel foam with a coated area of ~1 cm^2^, which were then pressed into slices under a pressure of 2 MPa for 30 s. Finally, the as-prepared working electrodes were completely dried in a vacuum oven for the following electrochemical tests.

The three-electrode setup was built by adopting 2 M of KOH as an aqueous electrolyte and employing an active material-loaded nickel slice, Hg/HgO electrode, and Pt wire as the working, reference, and counter electrodes, respectively. Cyclic voltammetry (CV) and galvanostatic charge/discharge (GCD) investigations were taken with the potential window from −1 to 0 V. The specific capacitance and Coulombic efficiency of electrodes in the three-electrode system were computed based on GCD data and the following two equations:

C = I·t_d_/ΔV·m (1)

η = t_d_/t_c_ (2)

where C, η, I, t_d_, t_c_, ΔV, and m stand for specific capacitance, Coulombic efficiency, discharge current, discharge time, charge time, discharge potential window, and the mass of active material, respectively.

The two-electrode configuration was constructed as well by assembling a symmetric supercapacitor device. Briefly, two slices of circular electrodes loaded with almost the same amount of active material were moistened by the electrolyte (2 M of KOH), placed face to face, separated by NKK porous fiber paper, and then set into a homemade mold (Figure 1). The CV and GCD tests of such supercapacitor cells were surveyed with an output potential range from 0 to 1 V. Similar to the calculation in a three-electrode system, the specific capacitance and Coulombic efficiency for the supercapacitor cell were acquired using Equations (1) and (2), but the parameter m in Equation (1) should be the whole amount of active material within both positive and negative electrodes. In addition, the energy and power densities of the supercapacitor cell could be further deduced in terms of the two equations described as follows:

E = (C·V^2^)/7.2 (3)

P = 3.6E/t_d_ (4)

where E, P, C, V, and td represent the energy density, power density, specific capacitance, output potential difference, and discharge time, respectively. Moreover, the electrochemical impedance spectroscopy (EIS) of the supercapacitor cell was performed at open circuit voltage with the frequency ranging from 0.01 to 100,000 Hz.

## 4. Conclusions

In summary, porous α-Fe_2_O_3_ hollow rods/reduced graphene oxide composites were fabricated using hydrothermally synthesized MoO_3_ nanobelts as a sacrificial template. Benefiting from a unique nanoscale structure, porous hollow characteristics, a high specific surface area and the synergistic effect of two components, the optimized α-Fe_2_O_3_ HR/RGO-30 composite showed enhanced supercapacitive behaviors with the largest specific capacitance of 426.3 F g^−1^, and desirable rate capability and appreciable cyclic stability in three-electrode system, which was comparable or even rather superior to lots of Fe_2_O_3_-based electrode materials. Furthermore, a symmetric supercapacitor cell α-Fe_2_O_3_ HR/RGO-30//α-Fe_2_O_3_ HR/RGO-30 was developed, and it released the maximum energy density of 18.7 Wh kg^−1^ with satisfactory rate performance and possessed stable electrochemical properties with 83.2% capacitance retention after continuously charging/discharging at a high current density of 5 A g^−1^ for more than 10,000 cycles. Moreover, the approach presented in this work could be extended to the development of many other transition metal oxide/RGO composites with fascinating architectures and improved properties, thus accelerating the exploration of high-performance energy storage devices.

## Figures and Tables

**Table 1:** Comparison of supercapacitive properties for Fe_2_O_3_-based electrode materials in three-electrode system.

Electrode Materials	Electrolyte	Maximum Specific Capacitance	Rate Performance	Cyclic Performance	References
Porous α-Fe_2_O_3_ nanostructure	0.5 M Na_2_SO_3_	193 F g^−1^@1 A g^−1^	90 F g^−1^@5 A g^−1^	92%@2 A g^−1^ (1000 cycles)	[17]
α-Fe_2_O_3_ hollow nanoshuttles	1 M KOH	249 F g^−1^@0.5 A g^−1^	~90 F g^−1^@8 A g^−1^	93.6%@8 A g^−1^ (2000 cycles)	[20]
Mesoporous α-Fe_2_O_3_ nanowire	1 M KOH	330 F g^−1^@1 A g^−1^	99.6 F g^−1^@10 A g^−1^	87%@2 A g^−1^ (2000 cycles)	[21]
α-Fe_2_O_3_ nano-flakes	1 M Na_2_SO_3_	171 F g^−1^@0.5 A g^−1^	36 F g^−1^@3 A g^−1^	85%@1 A g^−1^ (1000 cycles)	[46]
Hollow and porous Fe_2_O_3_ microrods	0.5 M Na_2_SO_3_	213 F g^−1^@2 A g^−1^	120 F g^−1^@6 A g^−1^	88%@6 A g^−1^ (5000 cycles)	[47]
α-Fe_2_O_3_@Ag microboxes	1 M Na_2_SO_3_	701 F g^−1^@0.1 A g^−1^	254 F g^−1^@5 A g^−1^	80%@10 A g^−1^ (2000 cycles)	[29]
RGO/α-Fe_2_O_3_ composite	2 M KOH	469.5 F g^−1^@4 A g^−1^	132.4 F g^−1^@16 A g^−1^	88%@8 A g^−1^ (5000 cycles)	[48]
V_2_O_5_-doped α-Fe_2_O_3_ nanotubes	3 M KOH	183 F g^−1^@4 A g^−1^	~110 F g^−1^@5 A g^−1^	81.5%@1 A g^−1^ (200 cycles)	[43]
Fe_2_O_3_/carbon nanotube arrays	2 M KOH	248 F g^−1^@8 A g^−1^	204 F g^−1^@24 A g^−1^	89%@8 A g^−1^ (5000 cycles)	[49]
α-Fe_2_O_3_ nanotube@MnO_2_ nanosheet	3 M KOH	289.9 F g^−1^@1 A g^−1^	118.3 F g^−1^@5 A g^−1^	85.3%@1 A g^−1^ (1200 cycles)	[50]
Fe_2_O_3_ nanorods/silver nanowires	1 M Li_2_SO_4_	287.4 F g^−1^@0.67 A g^−1^	177.8 F g^−1^@2 A g^−1^	60%@2 A g^−1^ (5000 cycles)	[51]
Porous α-Fe_2_O_3_/graphene	1 M Na_2_SO_4_	343.7 F g^−1^@3 A g^−1^	182.1 F g^−1^@10 A g^−1^	95.8%@10 A g^−1^ (50,000 cycles)	[52]
α-Fe_2_O_3_/porous carbon	1 M H_3_PO_4_	372 F g^−1^@0.7 A g^−1^	294 F g^−1^@1.5 A g^−1^	82%@1.5 A g^−1^ (1000 cycles)	[23]
Fe_2_O_3_/multiwall carbon nanotube film	1 M Na_2_SO_3_	431 F g^−1^@5 mV s^−1^	~160 F g^−1^@200 mV s^−1^	65%@100 mV s^−1^ (500 cycles)	[53]
α-Fe_2_O_3_/carbon nanotube sponge	2 M KCl	296.3 F g^−1^@5 mV s^−1^	~100 F g^−1^@300 mV s^−1^	60%@100 mV s^−1^ (1000 cycles)	[25]
Ti-doped Fe_2_O_3_@PEDOT nanorod arrays	5 M LiCl	311.6 F g^−1^@1 mA cm^−2^	208.1 F g^−1^@8 mA cm^−2^	96.1%@100 mV s^−1^ (30,000 cycles)	[54]
C_3_N_4_/Fe_2_O_3_ hollow microspheres	2.5 M Li_2_SO_4_	260 F g^−1^@0.5 A g^−1^	87 F g^−1^@5 A g^−1^	92%@1 A g^−1^ (1000 cycles)	[55]
α-Fe_2_O_3_ HR/RGO-30 composite	2 M KOH	426.3 F g^−1^@1 A g^−1^	219 F g^−1^@20 A g^−1^	87.7%@10 A g^−1^ (10,000 cycles)	This work

## Data Availability

Data are contained within the article.
